# Gaining Insight into Exclusive and Common Transcriptomic Features Linked with Biotic Stress Responses in Malus

**DOI:** 10.3389/fpls.2017.01569

**Published:** 2017-09-13

**Authors:** Bipin Balan, Tiziano Caruso, Federico Martinelli

**Affiliations:** Dipartimento di Scienze Agrarie e Forestali, Università degli Studi di Palermo Palermo, Italy

**Keywords:** biotic stresses, Malus, meta-analysis, protein-protein interaction network, transcriptomics

## Abstract

Identifying key information in transcriptomic data is very important, especially when the “omic” study deals with plant responses to stresses in field conditions where a high number of variables and disturbing factors may affect the analysis. In this meta-analysis we collected 12 transcriptomic works in Malus in order to identify which key genes, proteins, gene categories are involved in general plant pathological conditions and those features linked with exclusive biotic stress responses. Those genes that are only related with molecular responses to pathogen attacks and those linked with other plant physiological processes were identified. A pipeline composed by pathway and gene set enrichment analysis, protein-protein interaction networks and gene visualization tools was employed. A total of 13,230 genes of the 12 studies were analyzed with functional data mining tools: 5,215 were upregulated, 8,015 were downregulated. Gene set enrichment analysis pointed out that photosynthesis was inhibited by *Erwinia amylovora* and fungal pathogens. Different hormonal crosstalk was linked with responses to different pathogens. Gibberellin-related pathways, ABA-related were mostly repressed by fungal pathogens. Relating to transcription factors, genes encoding MYBs and WRKY2 were downregulated by fungal pathogens and 12 WRKYs were commonly regulated by different biotic stresses The protein-protein interaction analysis discovered the presence of several proteins affected by more than one biotic stress including a WRKY40 and some highly interactive proteins such as heat shock proteins. This study represents a first preliminary curated meta-analysis of apple transcriptomic responses to biotic stresses.

## Introduction

Apple (Malus × domestica Borkh) is one of the most important fruit crops in the world, which is having highly nutritional value and is strongly recommended in diet. There are several constrains which affect the cultivation of the apple trees, which can be classified as biotic stress and abiotic stress. The infections due to bacteria, fungus, and virus are severely affecting apple production and threatening grower's profits. Transcriptomic studies have been conducted in Malus (Chen et al., 2014; Kamber et al., 2016) as well as in other crops (Martinelli et al., 2012, 2013; Giovino et al., 2016) in order to shed lights on the complex molecular mechanisms of plant-microbe interactions. The identification of important proteins with a key role in gene-gene and protein-protein interaction networks is extremely useful to improve early diagnosis and therapeutic and genetic resistance strategies.

A comparison of the molecular mechanisms behind different stress conditions allows the discovery of potential candidate genes involved in specific and exclusive plant biotic stress responses. It also allows gaining insight into general and common features linked with disease status. This permits obtaining an early alert of the plant pathological status and addressing the most sustainable management strategies. Considering that transcriptomic studies are performed only in one season, often with no biological replications and in one specific environment, the importance of performing meta-analysis is getting higher. The presence, of many transcriptomic studies using different techniques (RNA-seq, microarrays, cDNA libraries etc.) for each crop, allows to gain insight into common and specific genes, pathways, and functional gene categories associated with different pathogens and commonly modulated between environmental stresses. It is known how some genes are affected by multiple environmental factors, involved in different metabolic, physiological, developmental, and organ-specific processes. Indeed it is essential to compare transcriptomic data dealing with multiple research objects in order to determine the pattern of expression of each gene in different physiological processes. This will help filtering biotic stress responses from unspecific features related to multiple physiological conditions. In Malus, several transcriptomic studies have been conducted to elucidate important plant physiological and developmental processes such as tree and root architecture, development and morphology, flavonoid pathway, and fruit physiological disorders (Krost et al., 2013; Mellidou et al., 2014; Ferrero et al., 2015; Wang et al., 2015; Li et al., 2016).

RNA sequencing and analysis using Next Generation Sequencing (NGS) methods have enabled to understand the gene expression data in both quantitative and qualitative manner (Martinelli et al., 2015). In each study, the large quantity of obtained data makes very difficult the analysis and the identification of the role of each gene in the molecular networks. False positive results often occur due to the RNA-Seq method that needs validation with other quantitative gene expression methods. Also, there are some genes, which can be expressed in any physiological condition, which makes the conclusions often weak. The large number of transcriptomic works published in plants requires more meta-analysis studies that would identify common and specific features in relation of the high number of objective studies performed at different developmental and environmental conditions. This is due to several reasons. First, transcript amounts are highly affected by changing environmental conditions and gene expression is finely modulated by a high number of variables such as timing, environmental factors and experimental conditions, tissues and their developmental stages, genotypes. Secondly, transcriptomic studies are often performed only one time with no repetition. Field studies are usually conducted only in one season leading to unreliable results affected by a high number of environmental disturbing factors. Third, few replicates (frequently only three) are usually considered due to the high costs of “omic” analysis. More biological replicates would be really useful to reduce environmental confounding variability. Fourthly, transcriptomic studies should be integrated with proteomics and metabolomics performed on the same samples of the same study in order to clarify post-transcriptional and post-transductional regulation mechanisms. Finally the identification of commonalities between similar independent studies to study the same factor would allow to identify which genes are more strongly associated with the subject of the study and focus the functional analysis only on those common findings. A clear discordance between different omic levels is usually observed in integrated approaches due to the fine-tuned molecular mechanisms of gene regulation developed by cells. Transcriptomic findings often did not closely match with miRNAome, proteome and metabolome data. Despite these issues, the extreme progress obtained in the development of sophisticated machines for omic analysis has allowed researchers to generate “omic” data with low budget requirements. How is it possible to extract the most useful information from the huge amount of produced data? How complex has to be the experimental design of these studies to obtain meaningful and trustful information? “Omic” experiments should be considered reliable if replications in different seasons and environments are performed.

Thus, a meta-analysis of all the transcriptomic studies plays a vital role to select the most frequent and most significant differentially expressed genes (DEG) among the complete list of differentially regulated genes.

The aim of this meta-analysis study was to identify key genes and proteins involved in general plant pathological conditions and those involved in specific and unique pattern of biotic stress responses in Malus. We used a customized pipeline of meta-analysis that could be applied to gain insight into similar studies in other crops.

## Materials and methods

### Search strategy of published study identification for meta-analysis

**Figure 1:**
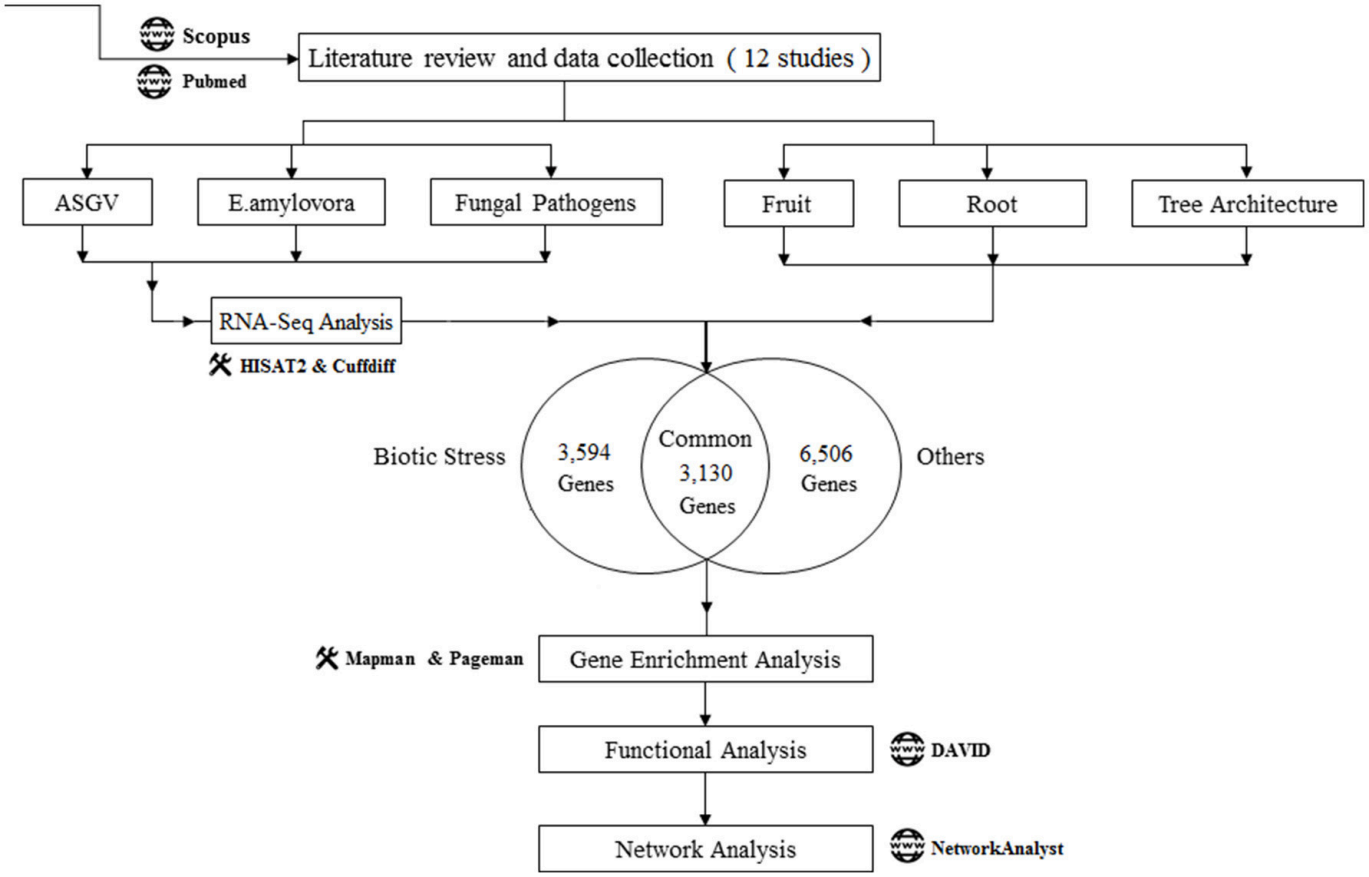
Work flow of the meta-analysis of the 12 Malus transcriptomic studies. Number of genes (upregulated and downregulated) uniquely modulated in biotic stress-related articles and in the rest of the studies were shown. Functional data mining tools were indicated.

The published transcriptomic studies in Malus x domestica were identified from Scopus and PubMed using the combination of keywords “Transcriptomics” and “malus” or “Transcriptomics” and “apple” in computer-based searches, and were published on or before March 2017. The identified studies were first divided into two major groups (1) “Biotic Stress” and (2) “Others”. For the “Biotic Stress” group, we considered the studies with the raw data are publically available. We found 12 studies related to our purpose of meta-analysis where six articles related with “Biotic Stress” and the rest six articles in the “Others” group. We downloaded the Raw data of all the “Biotic Stress” group studies and performed RNA-Seq analysis using a single analysis pipeline to obtain the differentially expressed genes. During the meta-analysis, we eliminated the common genes present in the two groups to get more accurate results pertaining to the objective of the study. According to the type of the pathogen, Biotic Stress studies were further divided into three groups (a) ASGV, (b) *Erwinia amylovora* (*E.amylovora*) (c) Fungal pathogens. Also, rest of the studies were divided into three groups: (d) “Tree Architecture” (e) “Fruit” (f) “Root.” The complete work flow of this meta-analysis was given in Figure 1.

### Differentially expressed gene selection and annotation

The Malus × domestica genome v1.0 and annotation file were downloaded from Phytozome (https://phytozome.jgi.doe.gov). The Raw files (SRA format) of the six articles dealing with biotic stress responses in Malus were downloaded from NCBI SRA and then converted to FASTQ format using SRA toolkit version 2.3.5. The article Gusberti et al. (2013) contains the differential gene expression information related to “Biotic Stress” and “Leaf development” in Malus and we downloaded the RAW files only for the samples dealing with “Biotic Stress.” The Raw reads were filtered to obtain high-quality clean reads by trimming low-quality bases followed by adaptor sequence removal using cutadapt version 1.8.1. The pre-processed reads were mapped to the Malus x domestica genome v1.0 with HISAT2 version 2.0.5 (Kim et al., 2015) using default parameters. The identification of differentially expressed genes was performed using Cuffdiff algorithm in Cufflinks version 2.2.1 pipeline with default parameters. The up and down regulated gens obtained with fold change cutoff (log2 FC >1 or log2 FC <−1) and *p*-value < 0.05 were only considered for the meta-analysis (Table S2A). The Raw file information, alignment information, and up- & down-regulated gene information are provided in Tables S1, S2A.

The up and down regulated gens with fold change cutoff (log2 FC >1 or log2 FC <−1) and *p*-value < 0.05 were collected from the rest six articles dealing with transcriptomic studies in Malus other than “Biotic Stress”(Table S2B).

All the differentially expressed gene ids was annotated using the Malus x domestica genome v1.0 mapping file downloaded from the Phytozome database (https://phytozome.jgi.doe.gov). The common and unique genes among different groups were identified. The common genes present in “Biotic Stress” and “Other” groups were eliminated from “Biotic Stress” gene list and were considered for the rest of the analysis. We have used custom made in-house perl script for the selection of gens and mapping.

### Gene enrichment and functional analysis

We used MapMan (Thimm et al., 2004) with the Malus x domestica mapping file (Mdomestica_196.txt) (http://mapman.gabipd.org/) to map the gene ids and visualize the metabolic overview, hormone regulation, transcription factors, and protein targeting of the Biotic stress gene sets (a) ASGV, (b) *E. amylovora*, (c) Fungal Pathogens.

The PageMan (Usadel et al., 2006) analysis plugin of MapMan was used to visualize differences among metabolic pathways using Wilcoxon tests, no correction, and an over-representation analysis (ORA) cutoff value of 3. We considered all the differentially expressed genes present in all 6 gene sets for the PageMan analysis: (a) ASGV (b) *E. amylovora* (c) Fungal Pathogens (d) Tree Architecture (e) Fruit (f) Root.

All the homologous TAIR IDs of the Biotic Stress genes were searched against the Database for Annotation, Visualization and Integrated Discovery (DAVID) version 6.8 (Huang et al., 2009) Web server (https://david.ncifcrf.gov/). We collected the unique list of TAIR IDs for each group and were used for the DAVID pathway analysis. The complete DAVID pathway search results for all six groups were given in Table S4. The gene ontology information related to Biological process were extracted (FDR cutoff = 0.05) from the DAVID result (**Table 2**).

### Protein-protein interaction network

Individual data annotation and analysis were performed using NetworkAnalyst (Xia et al., 2014), a web-based tool for protein–protein interaction network analysis and visual exploration. The unique list of homologous TAIR IDs of each “Biotic Stress” groups were uploaded and mapped against the STRING interactome database with default parameters (confident score cutoff = 900 and with experimental evidence) provided in NetworkAnalyst. We selected “Minimum Network” to simplify the network and to study the key connectivities. The common genes present in the three biotic stress groups (a) ASGV, (b) *E. amylovora*, (c) Fungal pathogens were highlighted in **Figure 5**. The genes present in each biotic stress groups and the common genes among them were highlighted separately in Figures S4, S5.

## Results

We collected a total of 12 transcriptomic studies published in Malus in Pubmed and Scopus databases and we compared the significantly regulated genes in each of these research subjects (*p*-value < 0.05, log2 FC >1 or log2 FC <−1; Table S2). The pattern of expression of the significantly regulated genes in each study and the common genes among the analyzed studies were shown in Table S3.

Article details, titles, analyzed tissues, and numbers of up- and down-regulated genes were listed in Table 1. The first six articles deal with biotic stress responses (Apple stem grooving virus, *E. amylovora*, and fungal pathogens) (Gusberti et al., 2013; Chen et al., 2014; Kamber et al., 2016; Shin et al., 2016; Yin et al., 2016; Zhu et al., 2017). The other six studies were dealing with the understanding of molecular mechanisms of fruitlet abscission, flesh browning disorder (physiopathological fruit disorder), flavonoid biosynthesis in fruit, tree and root architecture, growth and morphology (Krost et al., 2013; Mellidou et al., 2014; Ferrero et al., 2015; Petersen et al., 2015; Wang et al., 2015; Li et al., 2016). One study was divided in three datasets: responses to Venturia inaequalis in young and mature leafs and gene expression involved in leaf development (Gusberti et al., 2013). Although a great data variability was observed between the different studies regarding the number of significantly regulated genes, a strict *p*-value cut-off was kept in order to increase data reliability. Our meta-analysis workflow was shown in Figure 1. 13,230 genes were analyzed: 5,215 were upregulated, 8,015 were downregulated. Biotic stress-related works significantly regulated 5,218 while the rest of articles related to fruit processes, tree and root architecture and leaf development affected the expression of 8,012 genes. A part of these genes were commonly modulated (3,130). Among the two main categories (biotic stress and “others”), we independently analyzed subgroups of studies. Biotic stress was divided in responses to Apple Stem Grooving Virus, *E. amylovora* and fungal pathogens. The “others” group was divided in fruit processes, root and tree architecture. All these transcriptomic analysis were functionally mined with an integrated approach composed by gene set enrichment analysis (Pageman; Usadel et al., 2006), pathway and gene ontology analysis (DAVID; Huang et al., 2009), gene visualization (MAPMAN; Thimm et al., 2004), network analysis (NetworkAnalyst; Xia et al., 2014).

**Table 1:** Analyzed articles, objective of the studies, tissue number of up- or down-regulated genes, and assigned group.

**No**.	**Article**	**Objective**	**Tissue**	**Sample information**			**Pathogen**	**Group**
				**Total**	**Up**	**Down**		
1	Chen et al., 2014	Apple stem grooving virus	Shoot	667	263	404	Virus	ASGV
2	Kamber et al., 2016	Responses to *Erwinia amylovora*	Flower	255	147	108	Bacteria	*E. amylovora*
3	Yin et al., 2016	Resistance to Valsa mali	Twig	269	247	22	Fungi	Fungal pathogens
4	Zhu et al., 2017	Response to *Alternaria alternata*	Leaf	979	358	621	Fungi	
5	Shin et al., 2016	Response to *Pythium ultimum*	Root	1,278	355	923	Fungi	
6	Gusberti et al., 2013	Resistance to Venturia	Young leaf	1,318	751	567	Fungi	
			Mature leaf	452	244	208	Fungi	
		Leaf development	Young and mature leaf	5,744	1,655	4,089	–	Tree architecture
7	Krost et al., 2013	Tree architecture	Shoot	1,012	315		697	–
8	Ferrero et al., 2015	Fruitlet abscission	Fruit	507	470	37	–	Fruit
9	Mellidou et al., 2014	Flesh browning disorder	Fruit	70	44	26	–	
10	Wang et al., 2015	Flavonoid content	Fruit	113	88	25	–	
11	Petersen et al., 2015	Root architecture	seed, shoot, leaf	383	215	168	–	Root
12	Li et al., 2016	Root growth	Root	183	63	120	–	

### Gene set enrichment analysis

Gene set enrichment analysis showed that photosynthesis was repressed at transcriptional level in two biotic stress-related studies while it was enhanced during leaf growth and development (Figure S1). The gene categories related to primary metabolism such as photosynthetic light reactions, calvin cycle, major carbohydrate metabolism were expressed in Tree architecture. Trehalose pathway induction was linked with modifications on Malus tree architecture. RNA processing were more generally expressed by *E. amylovora* while these genes were not affected in response to other biotic stresses. Indeed, *Alternaria alternata* drives more the upregulation of genes involved in hormone metabolism ethylene, biotic stress, and protein degradation signaling compared to the other pathogens whereas it repress protein synthesis. As expected, root architecture was highly linked with the induction of auxin signaling and responsive genes as previously reported (Overvoorde et al., 2010).

As well as it concerns, different hormone categories were linked with specific research subjects. Gibberellin-related pathways were inhibited by the attack of *Pythium ultimum*. Apple stem grooving virus mainly repressed jasmonic acid-mediated responses. Gene encoding key players in biotic stress responses were linked with modifications of shoot architecture as well as with attacks of *A. alternata, E. amylovora*, and Venturia. Abiotic stress-related genes were upregulated by tree architecture modifications while an increase of induced genes involved in redox detoxifying pathways (ascorbate, glutathione-s-transferase, thioredoxin,) and biodegradation of xenobiotics were related to leaf development.

As far as it concerns, different categories of transcription factors were exclusively linked to different studies. Basic helix-loop-helix transcription factors were expressed by *P. ultimum*, Zn C2-CO-like were enhanced by leaf development and Zn C2-DOF were inhibited in fruits. MADS-box, SNF7, and MYB-related were also induced by leaf development processes. Homeobox transcription factor family proteins were more downregulated by Valsa mali infections. The bZIP transcription factor family proteins were more expressed in Root.

It is worthy to notice that different receptor kynases were involved in different physiological processes: receptor kinases were mostly repressed in fruit architecture changes. In protein synthesis, process expressed more in virus as well as *P. ultimum* infections. Protein degradation repression was linked to shoot development. Leucine rich repeat XI repression was linked with root development. DUF26 category upregulation was clearly linked to tree architecture. Aminoacid transport induction was associated only with root architecture processes. As expected, major transport related (sugar, nitrate, sulfate, phosphate, and nucleotides transport) genes were induced by leaf development processes.

The aim of this work was to focus on the biotic stress responses in order to identify genes related to general mechanisms of plant responses to biotic attacks and genes specifically modulated by different types of pathogens (virus, bacteria, and fungal pathogens). Indeed, we visualize only those significantly regulated genes in each of the three pathogen groups eliminating those genes that were also affected by other physiological processes and related to unspecific plant responses. Although the list of fungal pathogen-regulated genes were higher than the other two types of pathogens and this may disturb the meta-analysis it is clear that specific pathogens and some gene categories were specific for each pathogen.

Anthranilate N-hydroxycinnamoyl flavonoid related genes were more induced by ASGV. Alcohol-dehydrogenases flavonoid-related genes, nucleotide-related genes were more induced fungal pathogens. AGSV repressed few specific genes involved in phenylpropanoids, aminoacids primary metabolism (TCA, lipids, carbohydrates) (Figure S2). A low amount of genes were commonly modulated by the different stresses. They were involved in photosynthesis, minor CHO, and phenylpropanoid pathways.

### Biological process enrichment analysis

DAVID software was used to identify which gene ontologies (biological process, cellular component, molecular function) were significantly affected by six groups of transcriptomic works (responses to apple stem grooving virus, E. amylovora and fungal pathogens, fruit responses, root morphology, and architecture and tree architecture) (Table S4). Those categories with a *p*-value < 0.1 were shown. Related to biological processes, only responses to fungal pathogens, tree architecture and root responses showed significantly modulated biological processes (FDR < 0.05). Fungal pathogens repressed DNA-templated transcription regulation, photosynthesis, and microtubule-based movement while tree architecture repressed ubiquitin-dependent protein catabolic process, intracellular protein transport, protein N-linked glycosylation via asparagine, protein import into nucleus, docking, ribosomal large subunit assembly and ribosome biogenesis (Table 2). The chlorophyll catabolic process and oxidation-reduction process showed more expression in Tree architecture studies. In root, transcriptomic studies showed a significant up and down regulation in auxin-activated signaling pathway and showed inhibition of transmembrane receptor protein tyrosine kinase signaling pathway.

**Table 2:** Significantly regulated biological processes in the analyzed transcriptomic studies (FDR < 0.05).

**Biological Process**							
**Group**	**Up/Down**	**GO ID**	**GO term**	**Count**	***p*****-val**	**Fold enrichment**	**FDR**
Fungal pathogens	Down	GO:0006355	Regulation of transcription, DNA-templated	90	1.95E-05	1.555852518	1.69E-03
	Down	GO:0015979	Photosynthesis	15	2.49E-05	3.981704692	3.79E-02
	Down	GO:0007018	Microtubule-based movement	12	1.11E-06	6.868440594	3.79E-02
Tree architecture	Up	GO:0055114	Oxidation-reduction process	64	1.46E-05	1.752317802	2.97E-02
	Down	GO:0006511	Ubiquitin-dependent protein catabolic process	50	3.99E-08	2.312224705	1.65E-03
	Down	GO:0006886	Intracellular protein transport	40	9.92E-07	2.319412969	6.99E-03
	Down	GO:0042254	Ribosome biogenesis	31	6.76E-06	2.443142217	1.56E-02
	Down	GO:0000027	Ribosomal large subunit assembly	14	4.95E-06	4.47646703	0.0073212
	Down	GO:0000059	Protein import into nucleus, docking	11	4.41E-06	5.862040158	6.62E-05
	Down	GO:0018279	Protein N-linked glycosylation via asparagine	8	4.21E-06	8.952934059	1.12E-02
	Up	GO:0015996	Chlorophyll catabolic process	6	1.02E-05	18.20767717	2.22E-02
Root	Down	GO:0009734	Auxin-activated signaling pathway	11	1.31E-08	12.74993734	1.09E-02
	Up	GO:0009734	Auxin-activated signaling pathway	9	8.24E-06	8.762684211	8.22E-03
	Down	GO:0007169	Transmembrane receptor protein tyrosine kinase signaling pathway	8	1.92E-06	13.55238095	1.62E-05

*Only those studies with at least an affected biological process were indicated*.

### Hormone-related pathways

**Figure 2:**
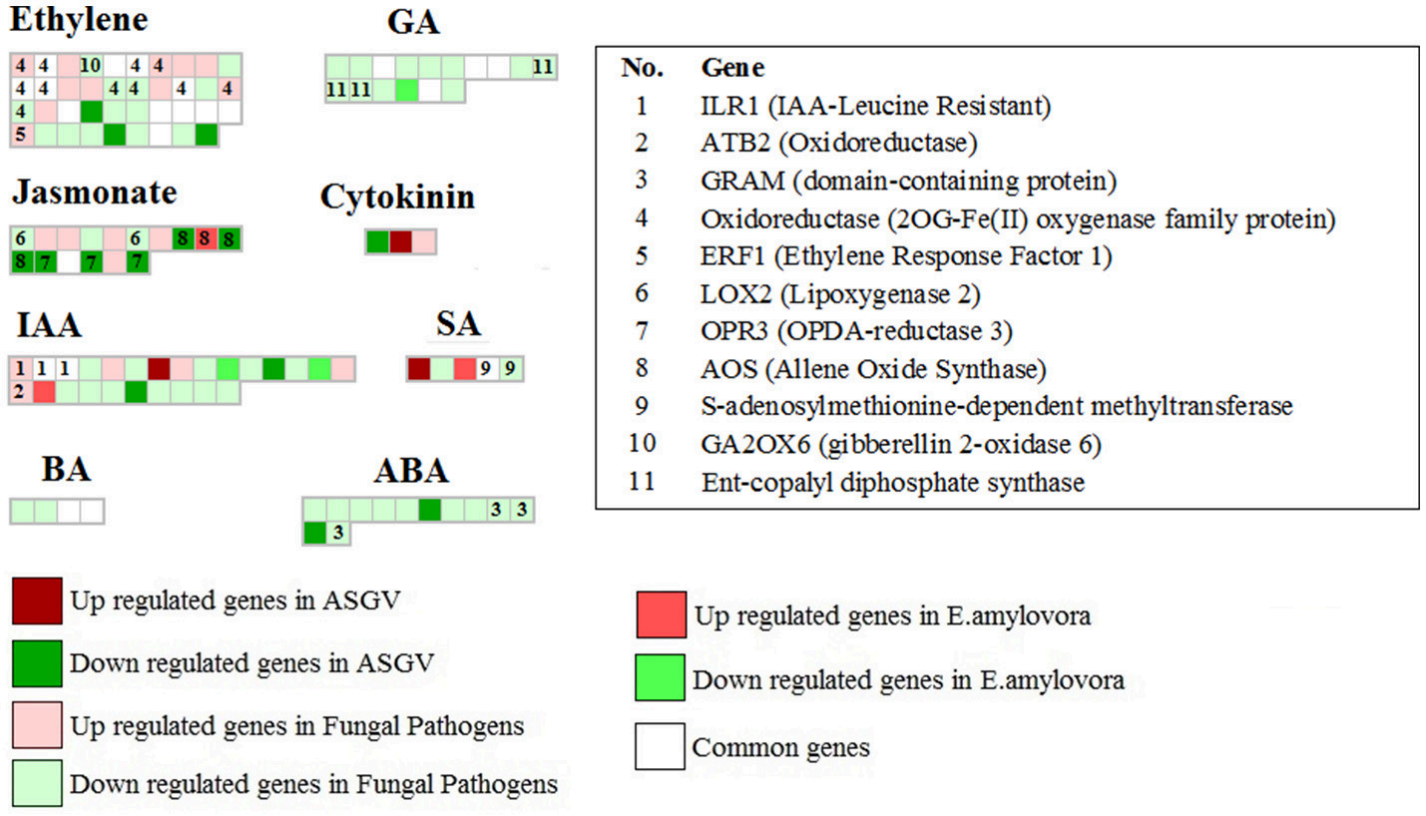
Gene expression changes involved in hormone-related pathways in response to Apple stem grooving virus, *Erwinia amylovora*, fungal pathogens, and commonly modulated in at least 2 of the 3 types of pathogens. Some key genes were indicated.

Infection of fungal pathogens enhanced expression of isoforms of *ILR1, ATB2*, and have opposite effects on the expression of different aldo/keto reductase (Figure 2). Two key brassinosteroid genes were downregulated by fungal pathogens and two were commonly modulated between different biotic stresses. Several genes involved in ethylene biosynthesis and signaling were enhanced by fungal pathogens such as *oxidoreductase 2OG-Fe(II) oxygenases* and ERF1. Many ethylene-related genes were commonly regulated by different biotic stress studies. Jasmonic acid-related genes were mostly repressed by apple stem grooving virus (*OPDA reductase3, allene oxide synthase*). *E. amylovora* enhanced the expression of a gene involved in salicylic acid response. Interestingly gibberellin-related and ARA-related genes were mostly repressed by fungal pathogens although four key GA-related genes were commonly modulated between different biotic stresses.

### Secondary metabolism

The expression of genes involved in secondary metabolism was peculiarly modulated by the different analyzed transcriptomic studies (Figure S3). Leaf development upregulated some genes of the non-MVA pathway (*CLA1, ISPF, CSB3*), it repressed other genes such as a zinc ion binding, a *GGPS1*, some 2-dehydro-3-deoxyphosphoheptonate aldolases and a shikimate synthase (Table S2). On the other hand transferases, a hydroxycinnamoyl-coa shikimate transferase, and a cinnamyl-alcohol dehydrogenase were enhanced by fungal pathogens. On the other hand *E. amylovora* induced a chalcone synthase. Several genes involved in dehydroflavonol and carotenoid pathways were upregulated during leaf development (Table S2).

### Protein targeting and transcription factors

**Figure 3:**
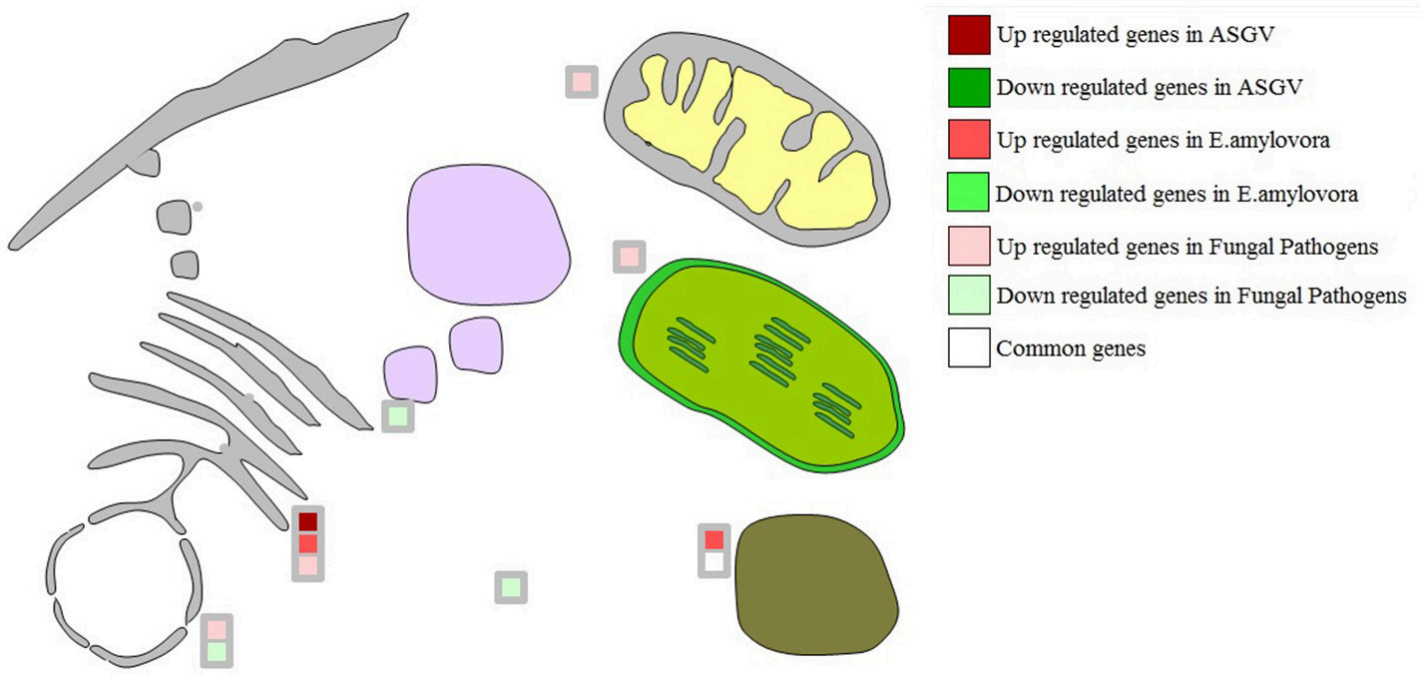
Gene expression changes involved in targeting-related genes in response to Apple stem grooving virus, *Erwinia amylovora*, fungal pathogens, and commonly modulated in at least 2 of the 3 types of pathogens.

**Figure 4:**
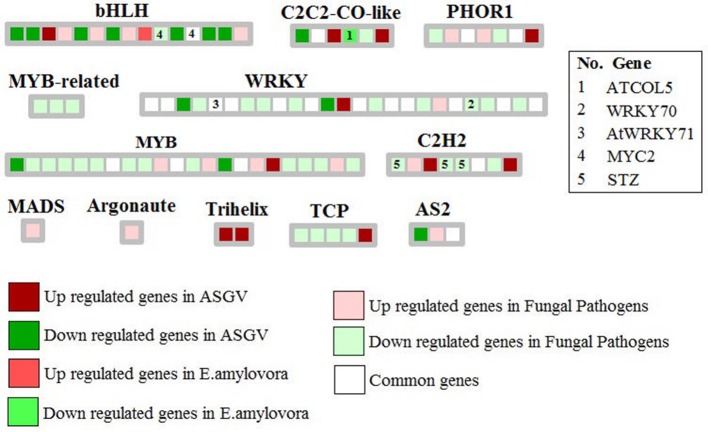
Significantly regulated transcription factor genes in response to Apple stem grooving virus, *Erwinia amylovora*, fungal pathogens, and commonly modulated in at least 2 of the 3 types of pathogens. Only few key TF categories were shown. Some key genes were also indicated.

Few key genes involved in secretary pathways differentially affected by the different biotic stresses (Figure 3). Nuclear transport factor 2 and VPS28-1 (vacuolar protein sorting-associated protein) were repressed by fungal pathogens while a signal peptidase subunit family protein was induced by *E. amylovora*. Specific transcriptomic changes were observed in relation to different types of pathogens. Fungal pathogens mostly inhibited MYB-related genes as well WRKYs and TCP transcription factors (Figure 4). Apple stem grooving virus inhibited seven genes encoding bHLH and two WRKY members while it induced two trihelix members (Figure 4; Tables S2, S3). *E. amylovora* induced one gene encoding bHLH and repressed one C2C2-CO-like gene.

### Protein-protein interaction network analysis

**Figure 5:**
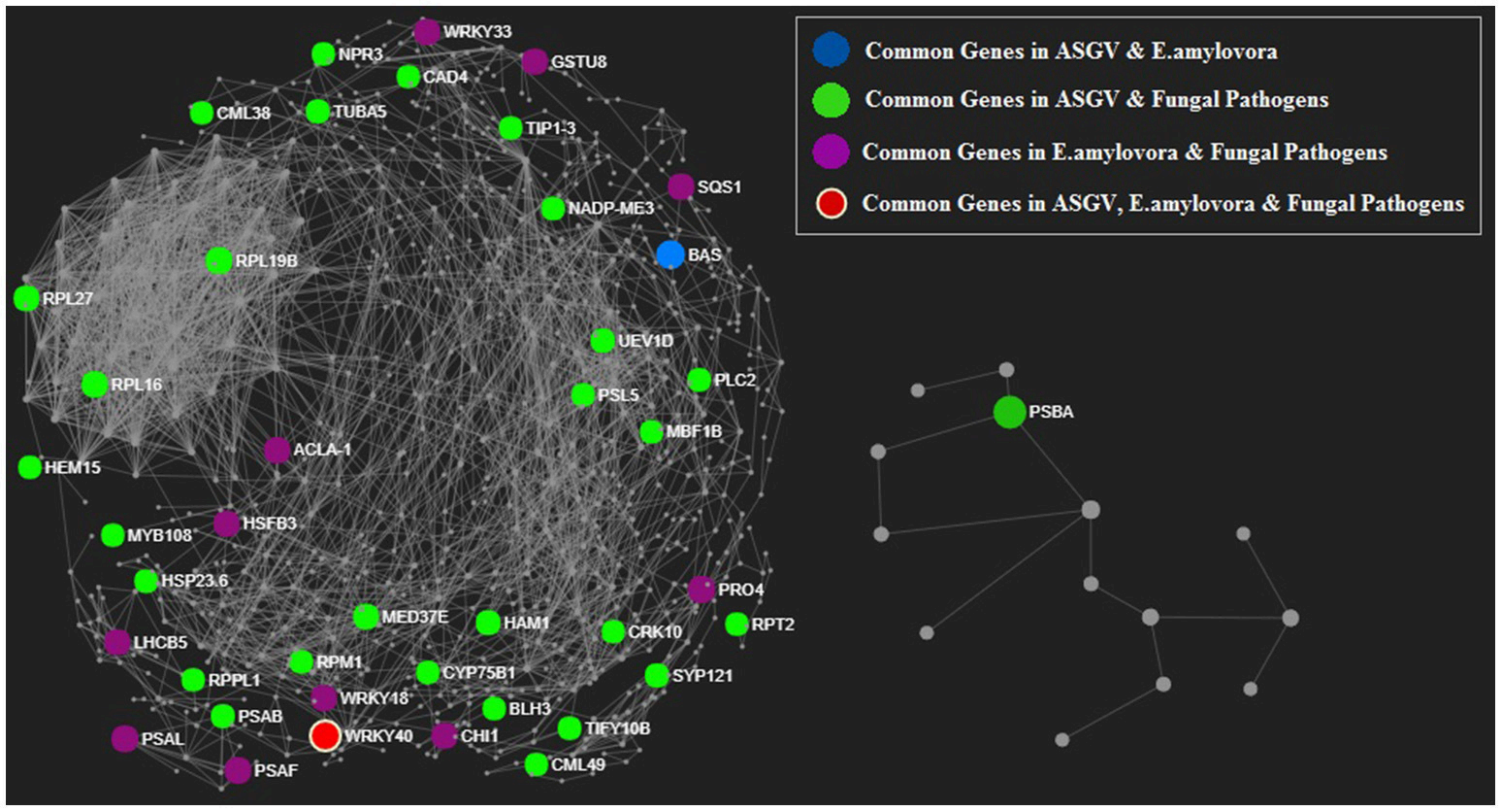
Protein-protein interaction network analysis predicted in Malus based on Arabidopsis knowledgebase. Proteins encoded by transcriptionally modulated genes were shown in different color basing on the type of pathogens. Proteins encoded by genes commonly modulated by 2 of 3 type of pathogens were shown in red.

A protein-protein interaction (PPI) network analysis was predicted in Malus based on Arabidopsis knowledgebase (Figure 5, Figures S4, S5). The list of biotic stress-related genes was determined. Those genes which were significantly affected in response to the three types of pathogens but not in the rest of transcriptomic studies were considered. Those biotic stress-related genes that showed to be modulated by other physiological processes were eliminated and considered to be unspecific. At the end, only the PPI network of the biotic stress-related genes and their partners were shown. The aim of this analysis was to identify some small interactive networks specific to each pathogens. GPA1, LPAT4 and XLG1 were closely connected and involved fungal pathogen responses (Figure S5). The protein network contain 28 proteins present in both ASGV and fungal pathogens, 11 proteins common between *E. amylovora* and fungal pathogens and one protein (BAS) in common between ASGV and *E. amylovora*. WRKY40 protein is the only one protein present in the network, which is present in all three biotic stress groups (Figure 5).

## Discussion

The large number of transcriptomic works published in plants really requires more meta-analysis studies that would identify common and specific features (genes, gene categories, pathways) linked with the different object of studies (plant developmental, agronomic, and environmental responses). Plant transcriptomic data are highly variable depending of different environmental conditions and gene expression is finely modulated by a high number of variables such as timing, genotypic differences, environmental factors and experimental conditions, tissues and their developmental stages. Here we have compared 12 transcriptomic studies in Malus in order to deliver functional genomic information linked with common or exclusive molecular responses to specific types of biotic stresses. In order to identify only those features related to biotic stresses we used also RNA-seq data related to other apple physiological processes. The aim was to perform a comparison analysis among transcriptomic datasets clarifying the role of key genes previously identified and shade lights on the different crosstalk played by important biological molecules such as hormones. Different transcriptomic studies are generally performed using different transcriptomic platforms and using different experimental design. In order to compare them, it is necessary to use at least the same bioinformatic pipeline. Meta-analysis in Malus is important to create a database of curated transcriptomic data that could be used also by the scientific community working on other crop species.

The repression of photosynthetic pathways at transcriptomic level in response to biotic stresses is a feature widely seen in previous transcriptomic studies (Martinelli et al., 2012, 2013). Here we showed that, among biotic stresses, fungal pathogens strongly inhibited primary metabolism genes. This evidence agrees with data obtained with imaging methodologies that analyzed chlorophyll and multicolor fluorescence. These published data demonstrated their possible application in improving early detection of infections of virus, bacteria, and fungi (Barón et al., 2016). The transcriptomic data our meta-analysis related to secondary metabolism confirmed findings obtained with imaging methodologies. The integration of different techniques is essential to drive pre-symptomatic stress detection. The reduction of photosynthesis has been observed in virus-infected leaves at symptomatic level (Pérez-Bueno et al., 2006; Pineda et al., 2010) and even before symptoms appear (Chaerle et al., 2007). Interestingly the integrated use of imaging and statistical analysis were used to determine infections of *P. syringae* in Arabidopsis before symptoms were visible (Berger et al., 2007). Fluorescence signals were also increased in sugar beet infected by powdery mildew and authors concluded that fluorescence indices could be considered as good indices of stress conditions (Leufen et al., 2014). Significant changes in photosynthetic activities linked with fungi infections are also spatially and temporally determined (Barón et al., 2016). Infections of bean leaves by rust fungi have been linked with changes in fluorescence induction kinetics (Peterson and Aylor, 1995).

Experimental algorithms have been developed to determine differences between affected and unaffected plants treated with phytotoxins of Alternaria brassicae (Soukupova et al., 2003). The use of molecular and phenotypic stress indicators would alloing manage pathogenesis and guiding effective management procedures of biotic stresses in plants. These techniques highlighted the role of pathogen in repressing photosynthetic performance and affect secondary metabolism. Indeed this let us to speculate that the integration of meta-analysis of transcriptomic works with the data obtained by techniques of chlorophyll measurements may improve both field and greenhouse management of plant diseases. A complementary use of molecular, remote sensing, and volatile sensor devices have shown to efficiently contribute in the early diagnosis of plant diseases and disorders (Dandekar et al., 2010). The use of these innovative integrated approaches represent the new frontier of plant pathology (Martinelli et al., 2015).

Auxins are considered the most regulator hormones of plant development (Taylor-Teeples et al., 2016). Lateral root development is one of the most well-known organogenesis process mediated by auxins. Auxin Response Factor (ARF) transcription factors are known to be key players in the auxin-mediated regulation of root development. Indeed ARFs have been found be repressed by the interactions with (Aux/IAAs) repressor proteins and the corepressor Topless. Proteins of the TIR1/AFBs bind auxins in a complex with the Aux/IAAs controlling phyllotaxy. AFB5, TIR1, F-box were upregulated on grafted apple and linked to root growth (Li et al., 2016) and they were not involved in the other studied physiological processes in Malus. This evidence highlighted their exclusive role in root development and growth. Our meta-analysis found out that, among the 12 analyzed Malus studies, root development processes uniquely induced GRAM-domain proteins. The GRAM domain has a length of 70 amino acids that is usually present in membrane-associated proteins and in glucosyltransferases (Doerks et al., 2000). Although some functions of these proteins remain unclear, the function of this domain seems to be linked with membrane-associated processes such as intracellular binding signaling pathways (Doerks et al., 2000).

Transcriptomic responses to *E. amylovora* (Kamber et al., 2016) showed that this pathogen is more linked with gibberellin response than the other studies as shown by the upregulation of four 2OG-Fe(II) oxygenase, *GA2OX6* and the repression of others (*GASA4* and unknown genes). The role of gibberellins in response to fire blight has been previously reported (Maxson and Jones, 2002). Indeed apple trees were treated with prohexadione calcium (Apogee) and trinexapac-ethyl (Palisade) well-known inhibitors of gibberellin biosynthesis. This work was showed to be effective in enhance resistance to *E. amylovora* (Maxson and Jones, 2002). This effect was mediated by a reduction of tree growth. However, in our meta-analysis we observed more GA-related genes modulated by fungal pathogens instead of E. amylovora. More studies are needed to define the role of gibberellins in plant responses to E. amylovora.

Interestingly jasmonic acid-mediated responses were generally repressed by leaf development process (Gusberti et al., 2013; Noir et al., 2013) and apple stem grooving virus infections (Chen et al., 2014). This latter evidence was expected since viruses are considered hemibiotrophic pathogen. Jasmonic acid (JA) and ethylene (ET) are critical for inducing immediate and effective responses against necrotrophs (Glazebrook, 2005) and they are usually repressed by Salicylic acid-mediated responses (Pieterse et al., 2009). Our meta-analysis highlighted that *A. alternata* infections showed to downregulate *LOX2* in a peculiar way (Zhu et al., 2017). *LOX2*, requires the expression of the F-box protein COI1 (CORONATINE INSENSITIVE1) that forms a ternary complex with JAZ repressor proteins (Zander et al., 2010). This let us to speculate that this might be detrimental for the infected Malus tree.

Two genes were commonly modulated by different biotic stresses agreeing with published literature confirming the important role of brassinosteroid in hormonal crosstalk in plants in responses to biotic stresses. Brassinosteroids have be known to be important player of biotic and abiotic stresses, although their mechanisms are still not well-elucidated. A homeodomain transcription factor OsBIHD1 is known to be involved in biotic and abiotic stress responses. The overexpression of this gene or its deficiency modulated the expression of several brassinosteroid-related genes causing brassinosteroid insensitivity (Liu et al., 2017). Indeed the function of this gene seems to modulate the trade-off between resistance and growth by regulating brassinosteroid-ethylene pathway (Liu et al., 2017). In addition it is worthy to notice that a squalene monoxygenase and squalene epoxidase3 were induced exclusively by Pythium infections among the 13 analyzed studies. On contrast a key positive regulator was repressed by apple stem grooving virus. These data agreed with previous data that showed how the silencing of a *N. benthamiana* squalene synthase, an important player of phytosterol biosynthesis, counteracted non-host resistance of *Pseudomonas syringae* and *Xanthomonas campestris*, increasing the growth of the host pathogen *P. syringae* pv tabaci by enhancing nutrient efflux into the apoplast. In addition, squalene epoxidase was induced in Calendula tropicalis by *Aspergillus niger* and this was linked with enhanced ginsenosides biosynthesis.

Extracting the data published by Gusberti et al. (2013) and dividing them in two datasets (one related to Venturia infection and one related to leaf development), we observed an increased expression of detoxifying pathways when leaves are developing and this implies that chemical defense pathways are induced during ontogenetic development against xenobiotic agents. As far as it concerns, different ontogenetic, development, and physiological process activated specific classes of transcription factors. This evidence could be helpful in elucidating the diverse gene regulatory networks modulating plant responses to different pathogen attacks. This will allow the development of specific strategy of genetic resistance to different pathogens.

Our meta-analysis showed that 12 genes encoding WRKYs were commonly modulated between different biotic stresses. Their modulation would be important to create genotypes resistant to the presence of multiple pathogens. Fungal pathogens mostly repressed WRKY genes implying that there might be a mechanism of repression of beneficial plant biotic-related genes. WRKYs represent a large family of transcription factors mostly found in plants with a key role in stress signaling among the several role where they are involved (Jiang et al., 2017). More than 100 and almost 200 WRKY superfamily members were discovered in *Glycine max* and *Oryza sativa*, (Rushton et al., 2010; Fan et al., 2015). Their expression is typically upregulated in plants when they are subjected to a great variety of stresses and they are activated by stress signals such as salicylic acid (SA) or other molecules. Their expression is rapid, transient and it is tissue-specific (Jiang et al., 2017). The identification of specific WRKYs modulated by different pathogens and abiotic factors would allow to address the genetic improvement to develop genotypes resistant to agronomical limiting factors. The complex network of protein-protein interactions may be visualized using software such as bioconductor package of R, Graphviz, Cytoscape. The main aim of this analysis to identify which highly interactive proteins are specifically or commonly modulated by each kind of the considered biotic stresses. Among them, we pointed our attention of WRKYs such as WRKY18, WRKY33, and WRKY40. Interestingly, the PPI network showed that WRKY40 was affected by all three kind of biotic stresses. Physical and functional interactions have een reported between WRKY18, WRKY40, and WRKY60 in response to pathogen infection in Arabidopsis thaliana (Xu et al., 2006). Our PPI network analysis confirmed the important role played by the interaction between WRKY18 and WRKY40 since these two genes were shown to be affected by both E. amylovora and fungal pathogens. It is well-known that these two WRKYs play an important role in PAMP-triggered basal defense (Pandey et al., 2010). These two WRKYs negatively affect EDS1 and PAD4, but positively upregulated some key JA-signaling genes.

In conclusion, our meta-analysis was effective in confirming the effects of fungal pathogen attacks on reduction photosynthesis at transcriptomic level highlighting the importance of integrating different molecular, imaging and high-throughput platforms in early diagnose of plant stress status. In addition it showed how specific hormones and transcription factor classes play specific roles in plant signaling responses to different pathogens. The PPI network highlighted the role of terpenoids in the response to pathogen attacks in Malus. The integrated meta-analysis approach and pipeline could be employed in comparing transcriptomic studies and deciphering common and exclusive features in the gene regulatory networks of other crop species.

## Author contributions

BB performed the meta-analysis using the described the functional genomic, bioinformatics tools. FM and TC conceived, designed the research work. FM mainly wrote the article. All authors significantly contributed on writing the manuscript.

### Conflict of interest statement

The authors declare that the research was conducted in the absence of any commercial or financial relationships that could be construed as a potential conflict of interest.
