# Supplementary material for: Gaining Insight into Exclusive and Common Transcriptomic Features Linked with Biotic Stress Responses in Malus
Source: Front Plant Sci. 2017 Sep 13;8:1569. doi: 10.3389/fpls.2017.01569 (PMC5601412; doi:10.3389/fpls.2017.01569)
Supplement: Supplementary file 1 [file Image1.PDF]

Supplementary Material

Gaining Insight into Exclusive and Common Transcriptomic Features Linked with Biotic Stress Responses in Malus

Bipin Balan[1], Tiziano Caruso[1], Federico Martinelli[1*]

[1]Dipartimento di Scienze Agrarie e Forestali, Università degli Studi di Palermo, Palermo, Italy.

*** Correspondence:**
Federico Martinelli
federico.martinelli@unipa.it

Supplementary Figures

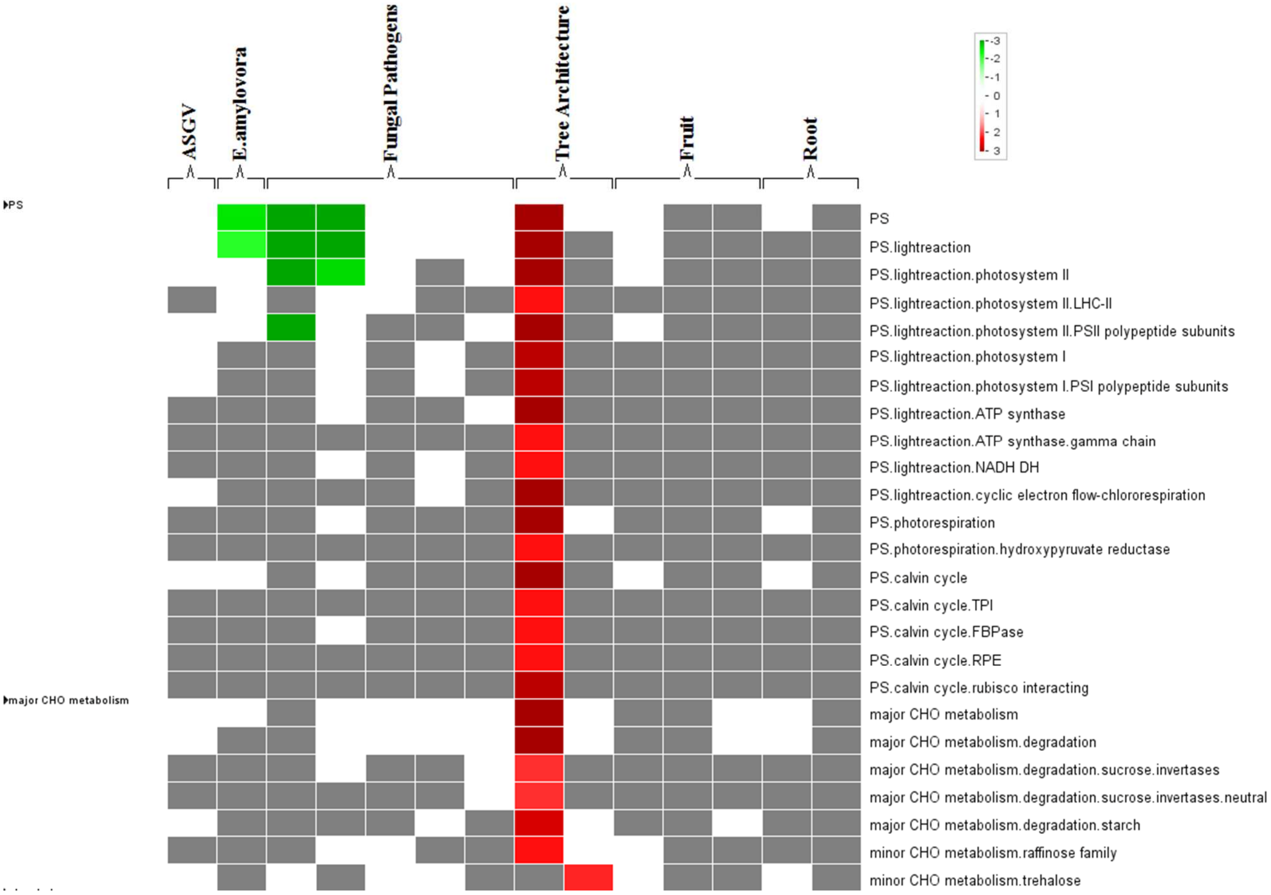

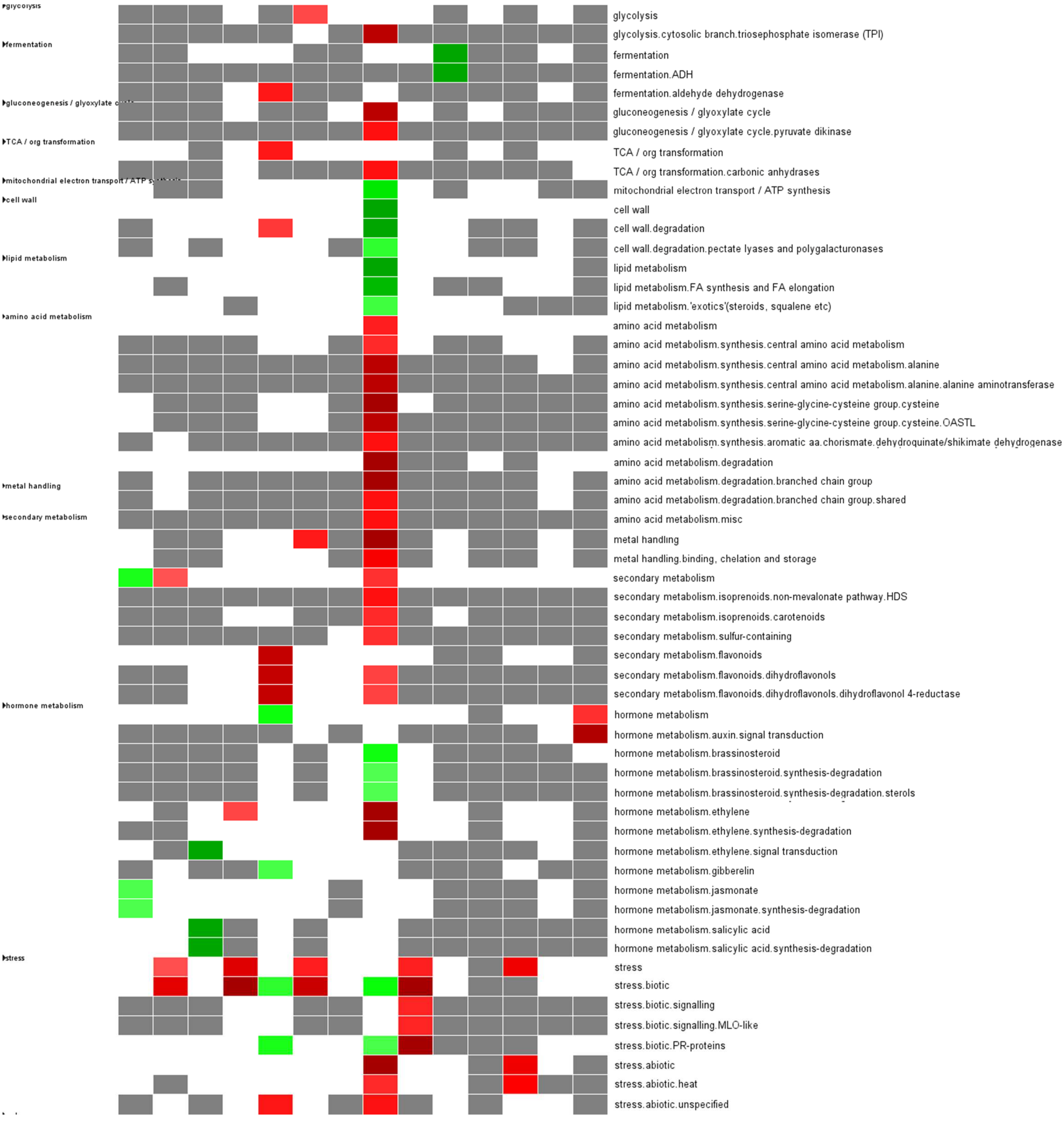
glycolysis
fermentation
TCA / org transformation
cell wall
lipid metabolism
amino acid metabolism
metal handling
secondary metabolism
hormone metabolism
stress
glycolysis
glycolysis.cytosolic branch.triosephosphate isomerase (TPI)
fermentation
fermentation.ADH
fermentation.aldehyde dehydrogenase
gluconeogenesis / glyoxylate cycle
gluconeogenesis / glyoxylate cycle.pyruvate dikinase
TCA / org transformation
TCA / org transformation.carbonic anhydrases
mitochondrial electron transport / ATP synthesis
cell wall
cell wall.degradation
cell wall.degradation.pectate lyases and polygalacturonases
lipid metabolism
lipid metabolism.FA synthesis and FA elongation
lipid metabolism.'exotics'(steroids, squalene etc)
amino acid metabolism
amino acid metabolism.synthesis.central amino acid metabolism
amino acid metabolism.synthesis.central amino acid metabolism.alanine
amino acid metabolism.synthesis.central amino acid metabolism.alanine.alanine aminotransferase
amino acid metabolism.synthesis.serine-glycine-cysteine group.cysteine
amino acid metabolism.synthesis.serine-glycine-cysteine group.cysteine.OASTL
amino acid metabolism.synthesis.aromatic aa.chorismate.dehydroquinate/shikimate dehydrogenase
amino acid metabolism.degradation
amino acid metabolism.degradation.branched chain group
amino acid metabolism.degradation.branched chain group.shared
amino acid metabolism.misc
metal handling
metal handling.binding, chelation and storage
secondary metabolism
secondary metabolism.isoprenoids.non-mevalonate pathway.HDS
secondary metabolism.isoprenoids.carotenoids
secondary metabolism.sulfur-containing
secondary metabolism.flavonoids
secondary metabolism.flavonoids.dihydroflavonols
secondary metabolism.flavonoids.dihydroflavonols.dihydroflavonol 4-reductase
hormone metabolism
hormone metabolism.auxin.signal transduction
hormone metabolism.brassinosteroid
hormone metabolism.brassinosteroid.synthesis-degradation
hormone metabolism.brassinosteroid.synthesis-degradation.sterols
hormone metabolism.ethylene
hormone metabolism.ethylene.synthesis-degradation
hormone metabolism.ethylene.signal transduction
hormone metabolism.gibberelin
hormone metabolism.jasmonate
hormone metabolism.jasmonate.synthesis-degradation
hormone metabolism.salicylic acid
hormone metabolism.salicylic acid.synthesis-degradation
stress
stress.biotic
stress.biotic.signalling
stress.biotic.signalling.MLO-like
stress.biotic.PR-proteins
stress.abiotic
stress.abiotic.heat
stress.abiotic.unspecified

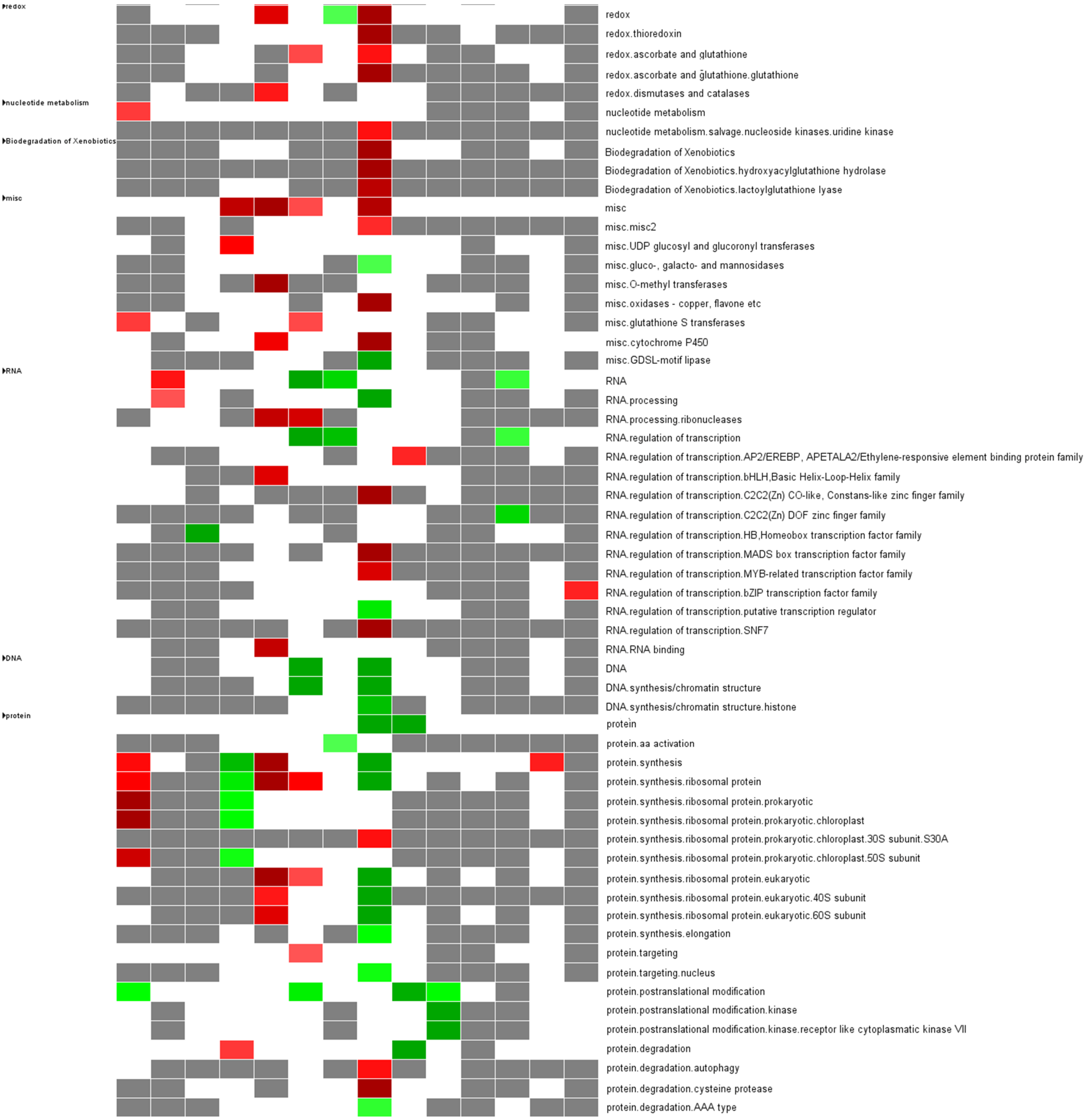
redox
nucleotide metabolism
Biodegradation of Xenobiotics
misc
RNA
DNA
protein
redox
redox.thioredoxin
redox.ascorbate and glutathione
redox.ascorbate and glutathione.glutathione
redox.dismutases and catalases
nucleotide metabolism
nucleotide metabolism.salvage.nucleoside kinases.uridine kinase
Biodegradation of Xenobiotics
Biodegradation of Xenobiotics.hydroxyacylglutathione hydrolase
Biodegradation of Xenobiotics.lactoylglutathione lyase
misc
misc.misc2
misc.UDP glucosyl and glucoronyl transferases
misc.gluco-, galacto- and mannosidases
misc.O-methyl transferases
misc.oxidases - copper, flavone etc
misc.glutathione S transferases
misc.cytochrome P450
misc.GDSL-motif lipase
RNA
RNA.processing
RNA.processing.ribonucleases
RNA.regulation of transcription
RNA.regulation of transcription.AP2/EREBP, APETALA2/Ethylene-responsive element binding protein family
RNA.regulation of transcription.bHLH,Basic Helix-Loop-Helix family
RNA.regulation of transcription.C2C2(Zn) CO-like, Constans-like zinc finger family
RNA.regulation of transcription.C2C2(Zn) DOF zinc finger family
RNA.regulation of transcription.HB,Homeobox transcription factor family
RNA.regulation of transcription.MADS box transcription factor family
RNA.regulation of transcription.MYB-related transcription factor family
RNA.regulation of transcription.bZIP transcription factor family
RNA.regulation of transcription.putative transcription regulator
RNA.regulation of transcription.SNF7
RNA.RNA binding
DNA
DNA.synthesis/chromatin structure
DNA.synthesis/chromatin structure.histone
protein
protein.aa activation
protein.synthesis
protein.synthesis.ribosomal protein
protein.synthesis.ribosomal protein.prokaryotic
protein.synthesis.ribosomal protein.prokaryotic.chloroplast
protein.synthesis.ribosomal protein.prokaryotic.chloroplast.30S subunit.S30A
protein.synthesis.ribosomal protein.prokaryotic.chloroplast.50S subunit
protein.synthesis.ribosomal protein.eukaryotic
protein.synthesis.ribosomal protein.eukaryotic.40S subunit
protein.synthesis.ribosomal protein.eukaryotic.60S subunit
protein.synthesis.elongation
protein.targeting
protein.targeting.nucleus
protein.postranslational modification
protein.postranslational modification.kinase
protein.postranslational modification.kinase.receptor like cytoplasmatic kinase VII
protein.degradation
protein.degradation.autophagy
protein.degradation.cysteine protease
protein.degradation.AAA type

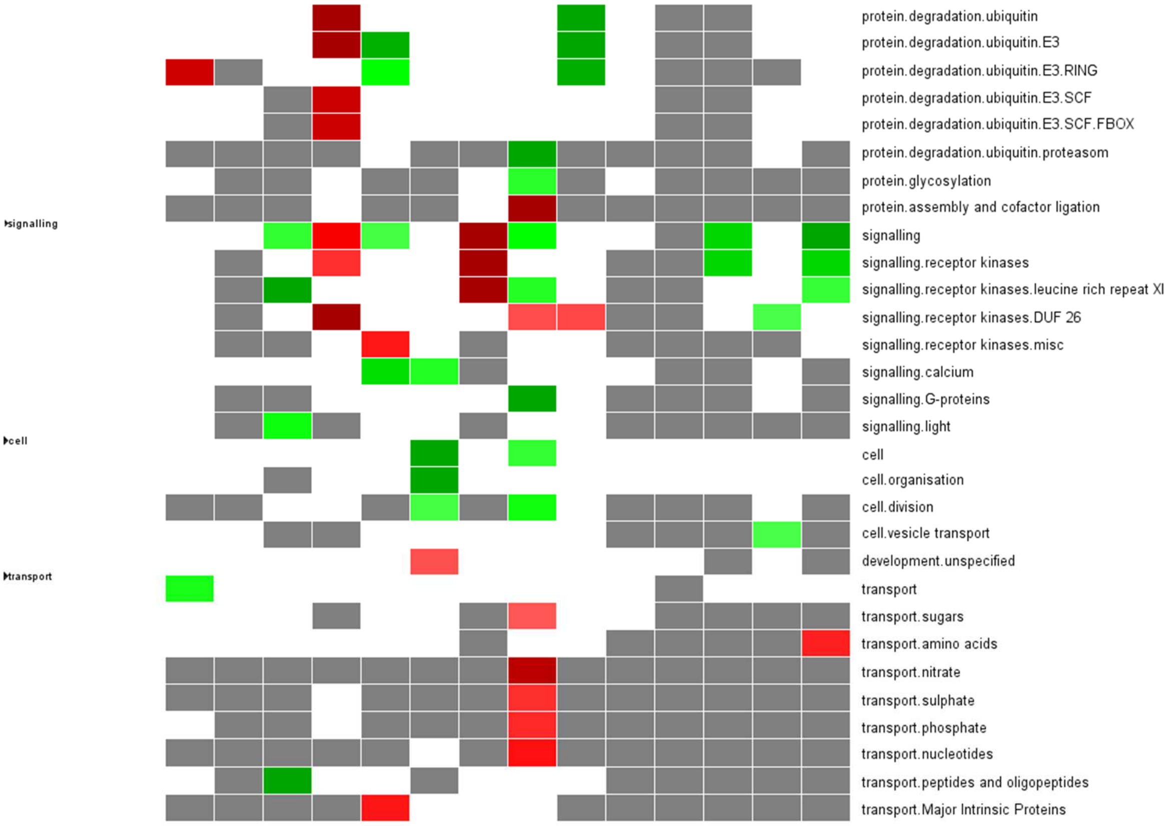

Fig. S1. Pageman analysis of the 14 transcriptomic datasets. Gene categories significantly modulated basing on Wilcoxon test (with no correction) were shown in green (downregulated) and red (upregulated).

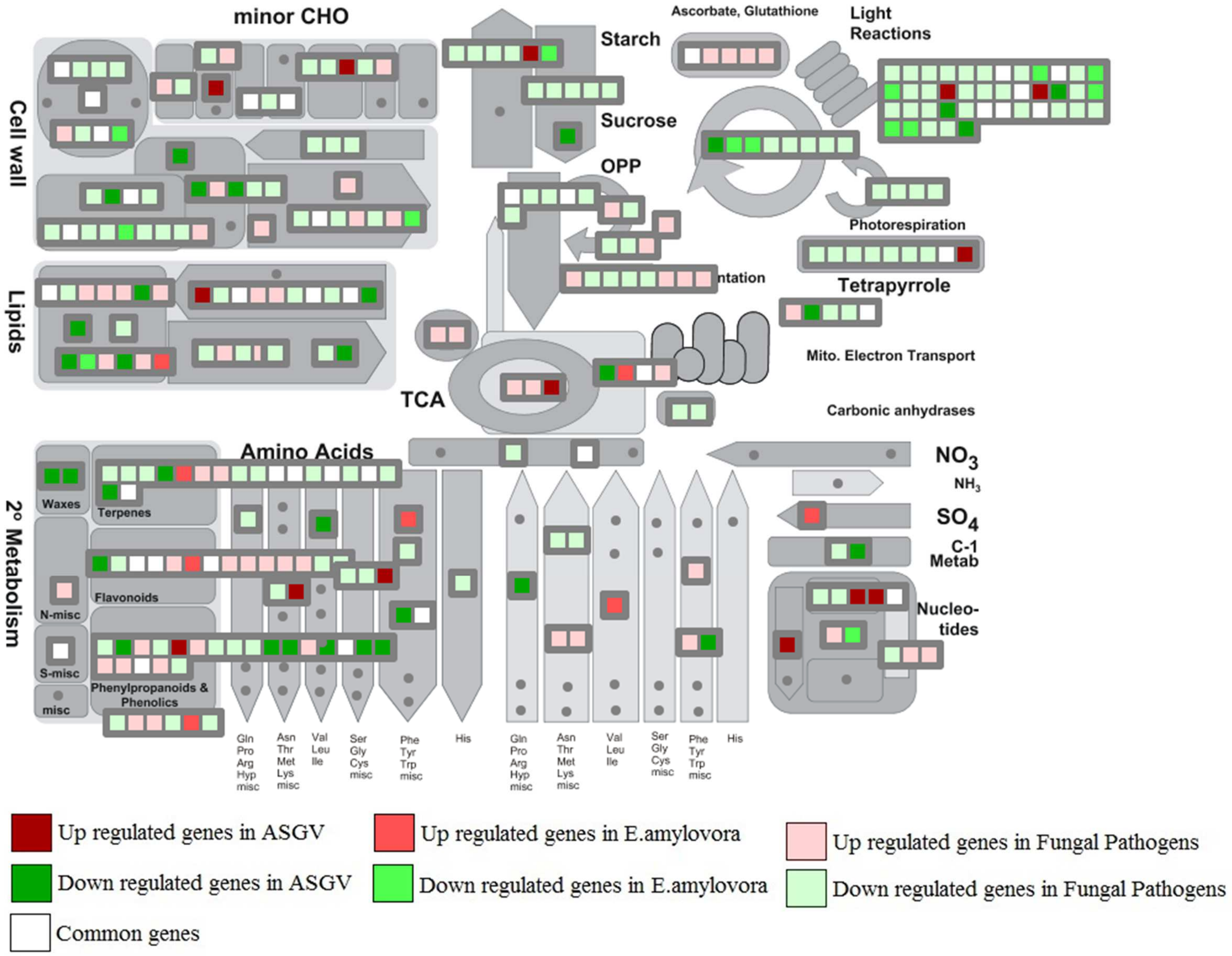

Fig. S2. Mapman overview of biotic stress-related genes in response to apple stem grooving virus (ASGV), Erwinia amylovora, fungal pathogens and commonly regulated in at least 2 of 3 types of pathogens. Only those genes that were uniquely modulated by biotic stress were indicated. Those affected in at least one of the rest of the transcriptomic studies were eliminated.

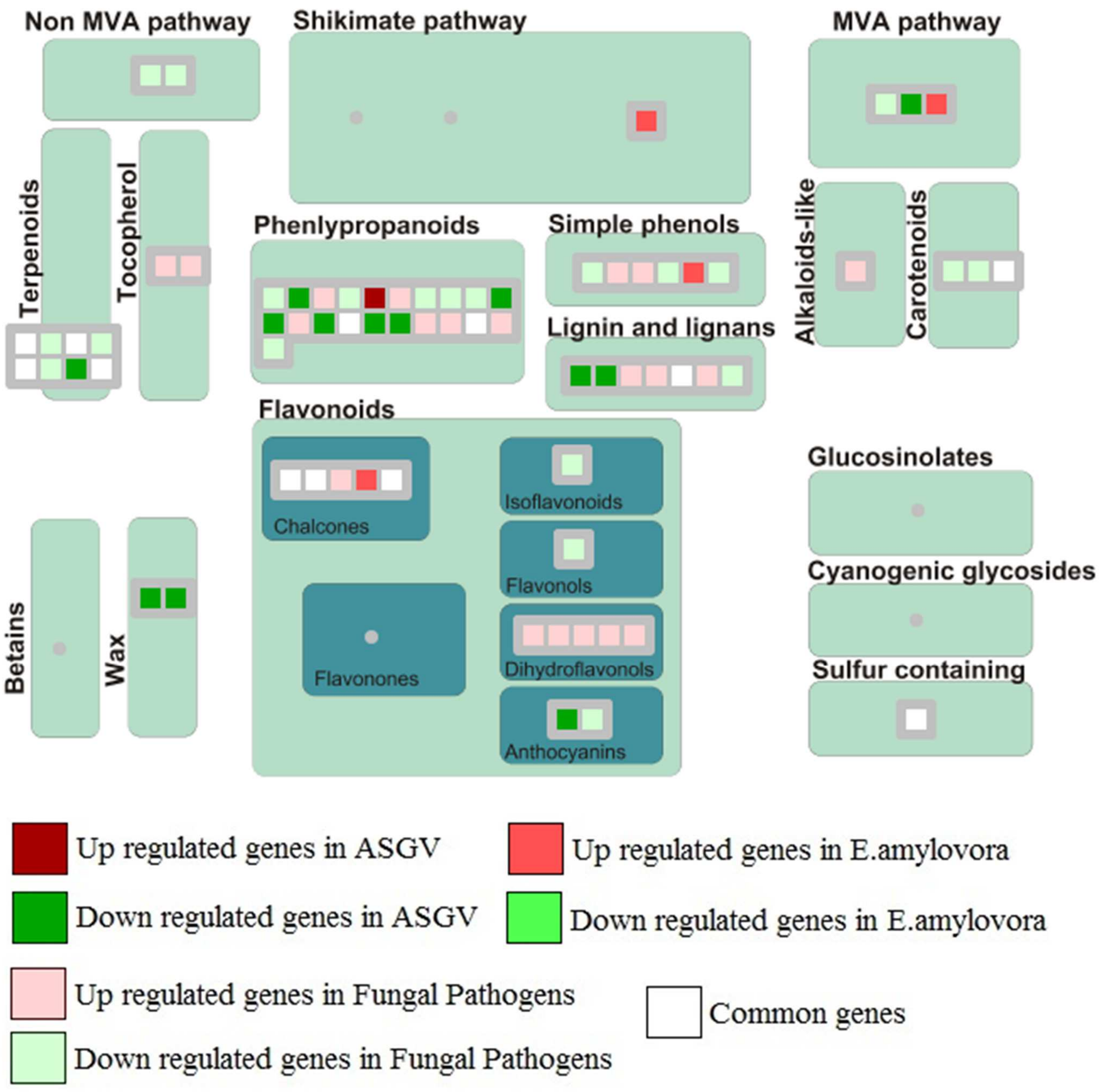

Fig. S3. Genes significantly regulated in biotic stress responses and involved in secondary metabolism and grouped based on exclusively or commonly expression in response to the three types of pathogen attacks.

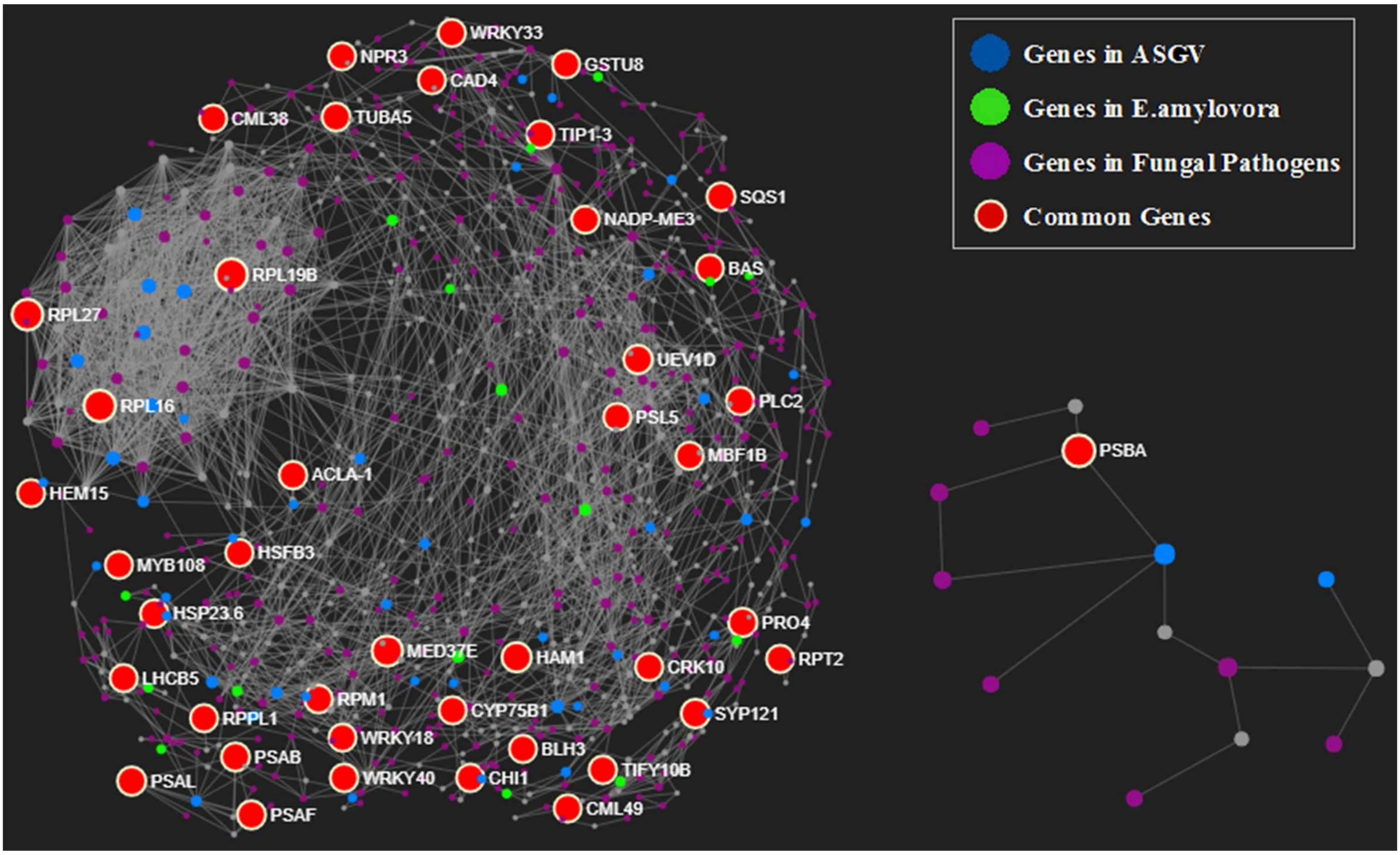

Fig. S4. Protein-protein interaction network analysis predicted in Malus based on Arabidopsis knowledgebase. Proteins encoded by transcriptionally modulated genes were shown in different color basing on the type of pathogens. Proteins encoded by genes commonly modulated by 2 of 3 type of pathogens were shown in red.

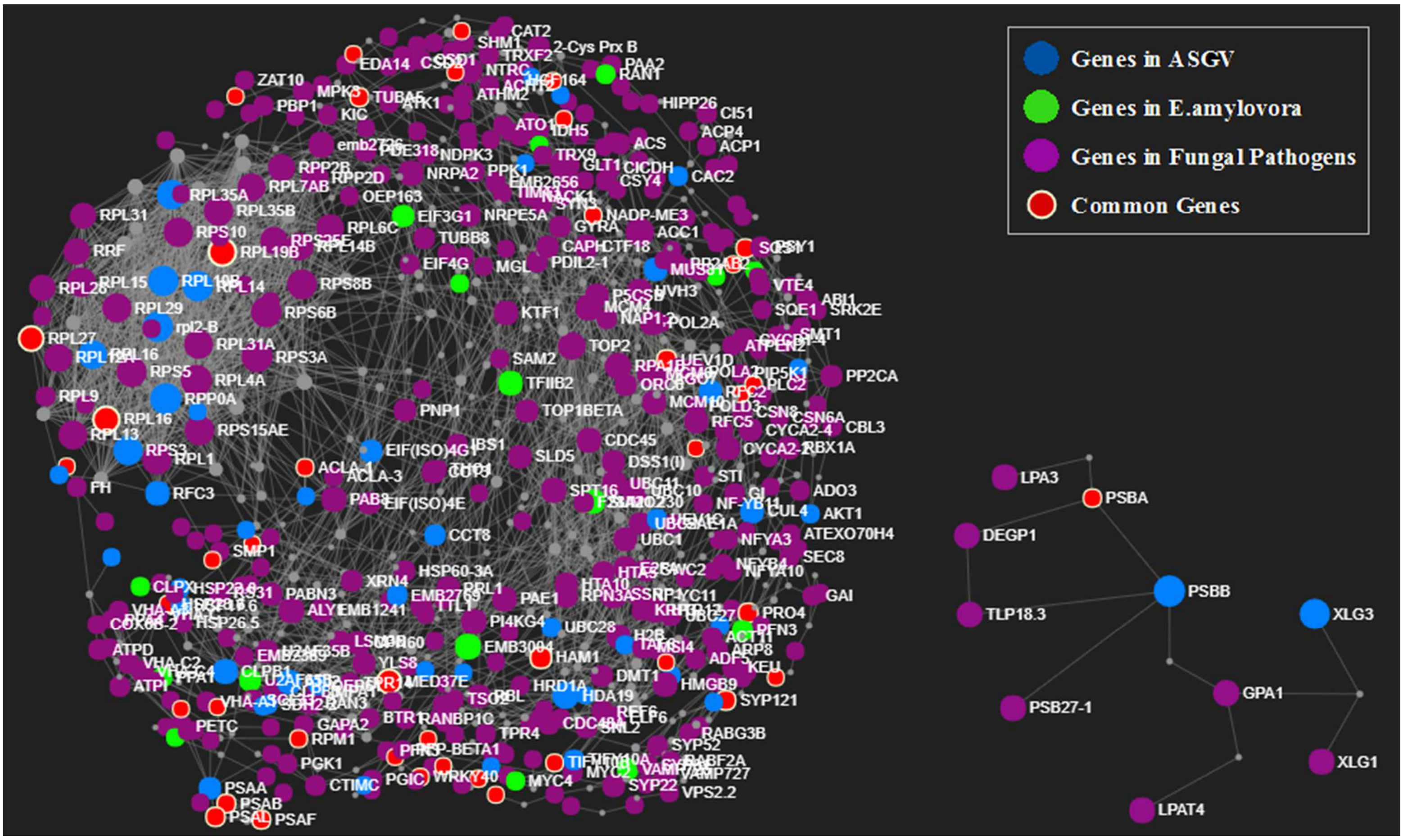

Fig. S5. Protein-protein interaction network analysis predicted in Malus based on Arabidopsis knowledgebase. Proteins encoded by transcriptionally modulated genes were shown in different color basing on the type of pathogens.
